# Combinatorial Fc modifications for complementary antibody functionality

**DOI:** 10.1080/19420862.2025.2465391

**Published:** 2025-02-14

**Authors:** Yannic C. Bartsch, Nicholas E. Webb, Eleanor Burgess, Jaewon Kang, Douglas A. Lauffenburger, Boris D. Julg

**Affiliations:** a Ragon Institute of Mass General, MIT, and Harvard, Cambridge, Massachusetts, USA; b Laboratory of Anti-Viral Antibody-Omics, TWINCORE-Institute for Experimental Infection Research, Helmholtz Center for Infection Research (HZI) and Medical School Hannover (MHH) and Cluster of Excellence RESIST (EXC 2155), Hannover, Germany; c Department of Biological Engineering, Massachusetts Institute of Technology, Cambridge, MA, USA

**Keywords:** Broadly functional antibodies, complimentary combinations, Fc-modifications

## Abstract

Therapeutic monoclonal antibodies (mAbs) can be functionally enhanced via Fc engineering. To determine whether pairs of mAbs with different Fc modifications can be combined for functional complementarity, we investigated the *in vitro* activity of two HIV-1 mAb libraries, each equipped with 60 engineered Fc variants. Our findings demonstrate that the impact of Fc engineering on Fc functionality is dependent on the specific Fab clone. Notably, combinations of Fc variants of the same Fab specificity exhibited limited enhancement in functional breadth compared to combinations involving two distinct Fabs. This suggests that the strategic selection of complementary Fc modifications can enhance both functional activity and breadth. Furthermore, while some combinations of Fc variants displayed additive functional effects, others were detrimental, suggesting that the functional outcome of Fc mutations is not easily predicted. Collectively, these results provide preliminary evidence supporting the potential of complementary Fc modifications in mAb combinations. Future studies will be essential to identify the optimal Fc modifications that maximize *in vivo* efficacy.

## Introduction

Numerous monoclonal antibodies (mAbs) are being developed to treat infections and malignancies.^1^ In addition to binding and neutralizing activity, antibodies, via the crystallizable fragment (Fc), can leverage Fc receptors (FcγR) on innate effector cells, leading to the recruitment of phagocytes, natural killer (NK) cells or deposition of complement; all functions that might be equally important for elimination of malignant or infected cells. Importantly, Fc-mediated activity can be precisely controlled by protein engineering as a versatile tool to improve biological activity. Such Fc modifications, however, are usually designed to enhance specific Fc-mediated functionalities by enhancing binding to dedicated Fc receptors.^2,3^ For example, Fc mutations SDIE and LPLIL have been used to specifically enhance FcyR3a binding and drive antibody-dependent cellular cytotoxicity (ADCC) against HER2+ cancers and B-cell lymphomas.^4–6^ In other disease contexts, however, multiple effector functionalities may be necessary to drive a therapeutic effect.^7,8^ Multiple effector functions have been proposed in the prevention of SARS-CoV-2 or RSV infections and control of chronic HIV or viral hepatitis.^9–11^ In addition to inducing broad effector functions, mAb combinations with complementary epitope coverage will be necessary to target highly variable infections such as HIV.^12^ Combining mAbs with different Fab specificities and complementary Fc-mediated functions can also expand the functional repertoire of a combinatorial antibody therapy.

## Results

To investigate if antibodies equipped with distinct Fc modifications, either as single Fab or Fab combination variants, will demonstrate additive functionalities, we selected two HIV-1 envelope-specific monoclonal IgG1 antibodies, RI808 and RI10953, which bind to different epitopes of the envelope glycoprotein (Env).^13^ Both clones have previously been selected not only for their HIV-1 neutralizing activity but also for their ability to bind to and opsonize HIV Env present on the surface of infected cells. RI808 targets the V3-glycan region of Env and RI10953 targets the CD4 binding site. Prior studies have shown that these antibodies have distinct baseline functional properties^13^ (Supplementary Figure 1). For each antibody, 60 previously described Fc variants were produced using our high-throughput Golden Gate cloning platform and these were comprehensively mapped for their ability to induce antibody-dependent NK-cell activation (ADNKA), monocyte and neutrophil phagocytosis (ADCP and ADNP) or complement deposition (ADCD) *in vitro* ^14^ (Table 1). These assays have previously been used to approximate the ability of antibodies to activate effector cells or complement.^10,14,35–41^ The goal of our study was to dissect the effects of Fc modifications on individual functions to point to specific characteristics of Fc variants rather than to determine a more complex interplay of combination of multiple effector cells.

**Table 1.** Fc variant mutation and respective amino acid changes (adapted from^14.^).

Variant Name	AA Mutations	Reported Effect	Ref.
IgG1	Human IgG1	isotype	–
IgG2	Human IgG2	isotype	–
IgG3	Human IgG3	isotype	–
IgG4	Human IgG4	isotype	–
AAA	T307A/E380A/N434A	↑ FcRn	^3^
AA	T307A/E380A	↑ FcRn	^15^
A	E333A	↑ FcRn	^15^
D376V	D376V	↑ FcRn	^16^
DVNH	D376V/N434H	↑ FcRn	^16^
E333A	E333A	↑ ADCD, ↑ADCC	^17,18^
E345R	E345R	↑ ADCD	^19^
E380A	E380A	↑ FcRn	^3^
EANA	E380A/N434A	↑ FcRn	^3^
EFTEA	G236A/S267E/H268F/S324T/I332E	↑ ADCC, ↑ADCP, ↑ADCD	^20^
HFST	H268F/S324T	↑ ADCD	^20^
I332E	I332E	↑ ADCC ↑ADCP	^21^
IEGA	G236A/I332E	↑ ADCC ↑ADCP	^22^
IgG3RH	R435H	↑ protein A	^23^
IgG3RHFY	R435H/F436Y	↑ protein A	^24^
K326W	K236W	↑ ADCD, ↑ADCC	^17,18^
KWES	K236W/E333S	↑ ADCD	^25^
LALA	L234A/L235A	no FcγR binding	^26^
LPLIL	F243L/R292P/Y300L/V305I/P396L	↑ ADCC	^27^
MLNS or LS	M428L/N434S	↑ FcRn	^15^
N297Q	N297Q	no FcγR binding	^28^
N434A	N434A	↑ FcRn	^3^
P257I	P257I	↑ FcRn	^15^
PIQI	P257I/Q311I	↑ FcRn	^15,16^
QL	T250Q/M428L	↑ FcRn	^29^
Q LS	T250Q/M428L/N434S	↑ FcRn	^15,29^
RGY	E345R/E430G/S440Y	↑ ADCD	^19^
S324T	S324T	↑ ADCD	^20^
SAEAKA	S298A/E333A/K334A	↑ ADCC	^21^
SDIE	S239D/I332E	↑ ADCC ↑ADCP	^21,22^
SDIEAL	S239D/A330L/I332E	↑ ADCC, ↑ADCP	^21,30^
SDIEALGA	G236A/S239D/A330L/I332E	↑ ADCC, ↑ADCP	^31^
SDIEALYTE	S239D/A330L/I332E/M252Y/S254T/T256E	↑ ADCC, ↑ADCP	^21,32^
SDIEGA	G236A/S239D/I332E	↑ ADCC, ↑ADCP	^22,30^
SDIESA	S239D/S298A/I332E	↑ ADCC	^33^
SEHFST	S267E/H268F/S324T	↑ ADCD	^20^
SELF	S267E/L328F	↑ FcγRIIb binding	^34^
YTE	M252Y/S254T/T256E	↓ADCC, ↑ FcRn	^32^

**Figure 1.**
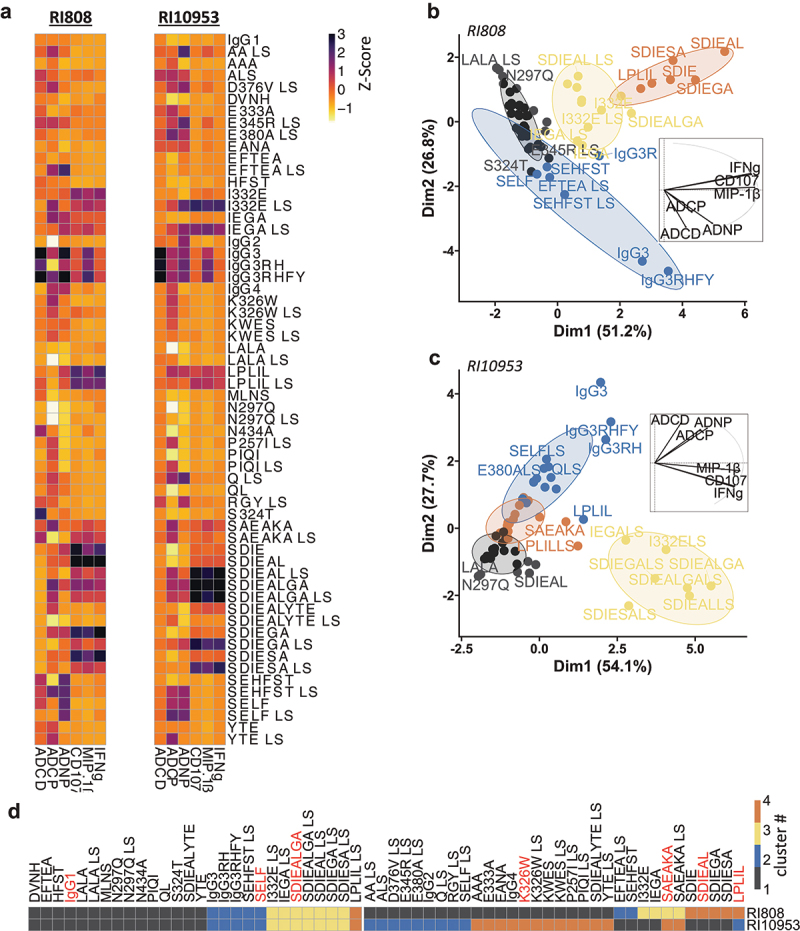
Functional characterization of the Fc-engineered antibodies mAbs RI808 and RI10953. Fc variants of the two clones were evaluated for induction of ADCP, ADNP, ADCD, NK cell degranulation (%CD107a+ of NK cells), NK cell secretion of IFNγ (%IFNγ+ of NK cells), and NK secretion of MIP-1β (MIP-1β+ of NK cells) after immune complex formation with recombinant HIV Envelope glycoprotein. (a) The heatmaps show the Z-Scored values of each individual readout (columns) and Fc variant (rows) for RI808 (left) and RI10953 (right). (b+c) Principal Component Analysis (PCA) of the variant data for RI808 (b) and RI10953 (c). Each point represents one variant (as labeled). Color and spheres show a k-means clustering (k = 4). Loadings plot (small plots within the PCA) indicate the contribution of the different Fc functions to separation in the PCA. (d) Comparison of the respective cluster each variant of each clone was assigned to by the k-means clustering.

Overall, and as expected, Fc activity was modulated in a mutation-dependent manner (Figure 1a and Supplementary Figure 2 and 3). For example, derivates of the SDIE (S239D/I332E) and LPLIL (F243L/R292P/Y300L/V305I/P396L) modifications were very potent in the induction of NK cell activation across the two mAb clones, while effector knockout mutations LALA (L234A/L235A) and N297Q, known to abrogate FcγR binding, had reduced Fc-mediated effector activity. To more comprehensively define the impact of the different Fc mutations, we performed a principal component analysis (PCA) followed by k-means clustering using Fc-mediated effector data for all Fc variants of the two Fab clones (Figure 1b + c). By using an empirical number of four clusters we obtained the best visual separation.

Among variants that shared cluster assignments across both clones, cluster 1 (black cluster) consisted of less functional variants, including the knockout N297Q and LALA variants. The observation that the nonmutated version (“IgG1”) of the two clones was also in this cluster highlights the strong potential of Fc-enhancing mutations. Clustering of IgG3 and its Fc derivatives, as well as the IgG1 SELF variant (“cluster 2”; blue), was driven by enhanced complement fixing and phagocytosis activity, whereas SDIE containing Fc mutations – characterized by enhanced NK cell activity – were grouped in a third cluster (“cluster 3”, yellow). The fourth cluster with little overlapping Fc variants between the two clones was not defined by the same functional features. In total, out of the 60 Fc variants per Fab clone, less than half (28 variants, ~47%) were assigned to the same cluster. As indicated by the loading plots, most variants were separated by NK cell activity or phagocytic and complement activity, creating a Fc activity gradient in the PCA. This may explain why some variants of the two clones were not grouped in the same cluster, given the stringent separation between two clusters along the gradient. For example, the LPLIL variant was clustered into cluster 4 for RI808 and cluster 2 for RI10953, although it increased NK activity for both clones, and was located at the border between clusters 2 and 4 for RI10953. Likewise, 21 RI808 variants were clustered in the nonfunctional cluster 1, whereas these modifications resulted in functional augmentation and distinct clustering for the respective RI10953 variants (Figure 1d). Thus, Fab-mediated binding may affect geometric properties of the Fc (i.e., angle and accessibility of the Fc) and how Fc mutations can influence interaction with Fc receptors.

In addition to modifications that alter binding to classical Fc receptors, we also included modifications that have been reported to extend serum half-life, such as MLNS, YTE, AAA, DVNH, or EANA, (Table 1) to analyze the effects on effector functions. Overall, and as expected, these mutations by themselves had modest or no influence on the elicited effector functions and were comparable to the unmutated IgG1. Given the clinical relevance of half-life-extending mutations, future mAb variants incorporating Fc modifications to enhance innate effector functions may also require extended half-life. Thus, we thought to combine a subset of Fc-altering variants with the MLNS variant (abbreviated LS in the combinations) on the same Fc. Although not the focus of our study, our initial data point to the potential interference of certain LS combined variants with Fc receptor binding and consequently effector functions (Supplementary Figure 4). Yet, it will be important to confirm and better characterize these findings more comprehensively in the future. Collectively, our data suggest that Fc engineering effects on Fc effector activity were comparable in a proportion of both clones, with introduction of distinct point mutations with potential to augment Fc activity, but that substantial clone-specific variations do exist.

**Figure 2.**
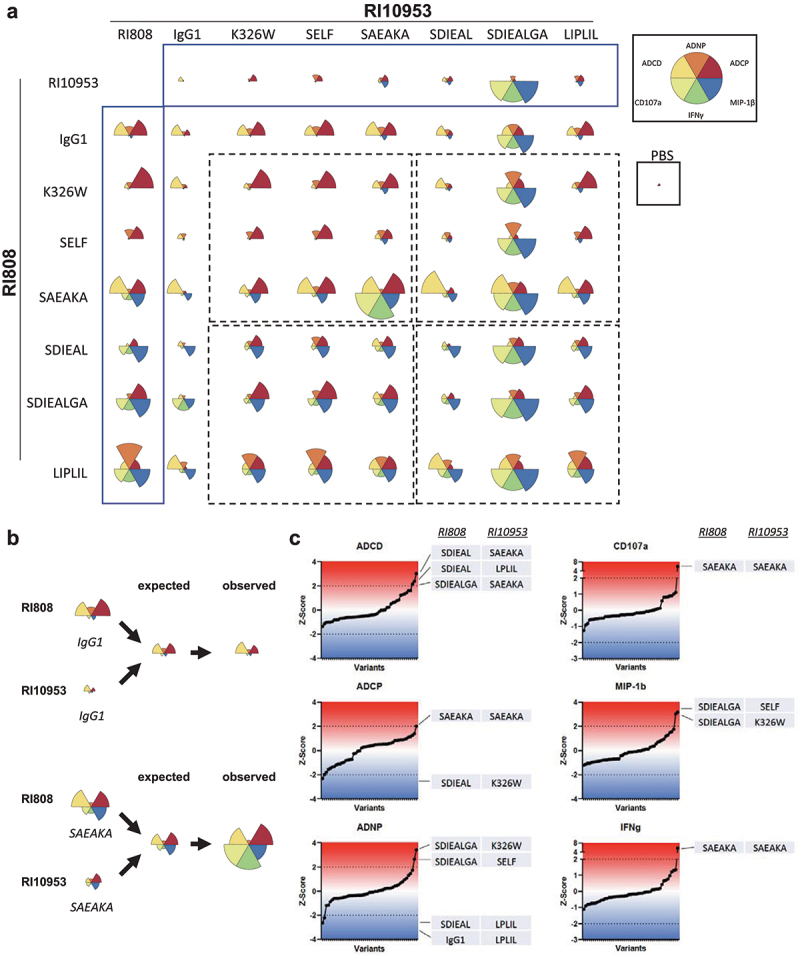
Heterologous combination of engineered mAbs can modulate Fc activity. (a) The flower plots summarize the functional activity of the respective combinations (variants of RI808 in rows with variants of RI10953 in columns; the first column and row represent the combination of the same variant of the same clone). Each petal represents the average of the Z-scored value for the indicated feature. Assay-specific total antibody concentrations for lone Fc variants (first column/row) were the same as the total concentration used for the combinations. (b) Visual representation of expected vs. observed functional breadth. To calculate the expected functional activity, we assumed that both variants would contribute with half of the respective activity that was observed when tested uncombined. Observed is the actual measured data after Z scoring. (c) We calculated the difference of observed and expected value per function. Shown is the Z-Score of this difference matrix (per individual function). Combinations with a difference outside 95% confidence interval (Z-Score >|2|) are indicated (left variant is clone RI808, right variant is clone RI10953).

While our data indicate that Fc engineering can efficiently enhance specific Fc-mediated effector functions, most of the variants are designed to preferentially target specific Fcγ receptors or C1q.^42^ To enhance functional breadth, we investigated potential synergistic effects of Fc variant combination across the two clones. To test our hypothesis that combination of two variants produce a combinatorial functional response, we selected six IgG1 Fc variants of both clones. For guided selection we first ranked each variant by their abilities to induce the respective functions (Tables 2 and 3) and calculated a “functional bias” score indicating whether a variant was more prone to induce phagocytosis (F-Score <0), NK cell activation (F-Score >0) or a balanced response (F-Score ~0) (Tables 2 and 3). We intentionally focused directly on Fc effector functions rather than FcγR binding to capture the integrated biological activity for each of the variants. In addition to the unmutated IgG1, K326W and SELF variants were included for their phagocytosis activity, while the SAEAKA variant was included as a primarily phagocytic variant that also retained some NK cell activity. SDIEAL, LPLIL and SDIEALGA were potent NK activating variants with some phagocytic activity. All selected variants were combined in pairs at equal concentration in all assays (Figure 2a and Supplementary Figure 5). Homologous combination (different Fc variants of the same clone) led mostly to a reduction of Fc activity, when compared to the single variant (at the same total concentration), indicating potential binding competition of the two Fc variants which would bind the same epitope. Only for combinations of RI10953 K326W, SELF and LPLIL, which alone showed little ADCP activity, we observed enhanced ADCP activity (Supplementary Figure 5). In contrast, heterologous combinations (Fc variants of different Fab clones) were overall more prone to enhance the overall functional activity (Figure 2a).

**Table 2.** Functional ranking of RI808 Fc variant.

		RI808					
*Variant*	ADCP		ADNP	CD107a	MIP-1β	IFNγ	*F-Score*
SDIEAL	10		8	1	1	1	−3.2
SDIESA	11		11	4	3	3	−2.4
SDIEGA	6		4	3	2	2	−2.0
SDIE	12		5	2	4	4	−1.9
LPLIL	23		6	5	5	5	−1.9
I332E	14		9	7	7	6	−1.3
N297Q	29		29	26	22	17	−1.1
LALA	26		27	15	14	12	−0.5
E333A	25		19	10	11	11	−0.4
MLNS	27		22	22	12	14	−0.4
SDIEALGA	1		7	6	6	7	−0.3
N434A	20		28	29	10	29	−0.1
DVNH	21		24	18	16	15	−0.1
EANA	17		25	16	27	22	0.0
EFTEA	24		16	19	20	19	0.1
AAA	15		23	28	29	28	0.2
S324T	22		14	13	18	21	0.2
IgG1	18		18	23	21	24	0.3
PIQI	7		26	11	23	16	0.3
HFST	16		17	24	24	18	0.4
SAEAKA	2		12	9	8	9	0.4
YTE	8		21	17	17	20	0.5
SDIEALYTE	5		20	12	15	10	0.5
KWES	13		10	20	25	26	0.7
QL	9		13	21	19	25	0.7
SEHFST	28		1	14	13	13	0.8
IEGA	3		3	8	9	8	0.8
K326W	4		15	27	26	27	1.1
SELF	19		2	25	28	23	1.4

Data of each functional readout was Z-Scored and each variant ranked by its function (1 most functional, 29 least functional). Rank is indicated in the table. To evaluate phagocytic vs. NK activating potential of each variant, a functional score (F-Score) was calculated (F-Score <0 NK prone response; F-Score >0 phagocytosis prone response; compare methods).

**Table 3.** Functional ranking of RI10953 Fc variant.

		RI10953					
*Variant*	ADCP		ADNP	CD107a	MIP-1β	IFNγ	*F-Score*
SDIEALGA	2		2	1	1	1	**−2.1**
SDIEAL	22		15	3	5	5	**−1.1**
SDIE	24		16	7	7	7	**−0.9**
SDIEALYTE	12		29	5	4	3	**−0.9**
N297Q	29		27	25	29	21	**−0.9**
LALA	28		28	16	22	25	**−0.8**
SDIEGA	21		13	8	9	8	**−0.7**
SDIESA	15		10	4	6	6	**−0.7**
PIQI	26		26	27	28	27	**−0.5**
QL	27		18	29	25	28	**−0.4**
DVNH	23		24	24	14	23	**−0.4**
N434A	25		19	26	20	29	**−0.4**
I332E	11		23	9	8	9	**−0.3**
IEGA	13		14	10	11	10	**−0.3**
S324T	16		21	11	15	12	**−0.3**
IgG1	17		22	22	16	13	**−0.3**
MLNS	18		17	17	18	14	**−0.2**
HFST	14		20	20	21	24	**−0.2**
SEHFST	20		12	21	26	20	**−0.1**
**EFTEA**	19		7	14	12	17	**0.0**
SAEAKA	1		6	6	3	4	**0.3**
AAA	9		25	28	23	26	**0.3**
YTE	10		9	19	27	22	**0.3**
LPLIL	4		3	2	2	2	**0.4**
KWES	7		11	13	17	18	**0.6**
EANA	8		4	15	10	11	**0.8**
K326W	6		8	18	19	16	**0.8**
E333A	5		5	12	13	19	**1.0**
SELF	3		1	23	24	15	**1.7**

To assess whether some heterologous combinations over- or underperformed relative to their uncombined counterparts in terms of Fc functionality, we compared the expected functional activity (based on the uncombined activity) to the actual observed activity (Figure 2b). Based on the difference (actual vs. observed), we ranked the variant combinations per Fc effector function and defined a Z-Score −2 and 2 as thresholds for under- and overperforming combinations, respectively. While most combinations were within these limits, some combinations had an increased functional activity (Figure 2c). For example, the heterologous combination of the SAEAKA variant of both clones elicited much higher ADNKA (CD107a expression and IFNγ secretion) and ADCP than expected and this combination had overall the highest functionality in our dataset (Figure 2c). Other overperforming combinations included RI808 SDIEAL/SIDEALGA and RI10953 LPLIL or SAEAKA of RI10953 for ADCD, or RI10953 K326W or SELF for ADNP and ADNKA (NK cell MIP-1β secretion). Thus, the combination of Fc variants of two non-competing clones can increase overall functional activity and breadth by not only broadening Fc receptor usage on effector cells but also by increasing the total amount of antibodies that can be immobilized on one antigen.

In contrast, in a subset of the tested Fc variant combinations (such as RI808 SDIEAL and RI10953 K326W in the ADCP assay), we observed dampening instead of enhancing activity. Phagocytes such as neutrophils or monocytes can express a range of different Fc receptors whereas NK cell activation is mostly limited to FcγR3a. Thus, Fc variants modulating affinity to Fc receptors other than FcγR3a may also negatively impact NK cell activation. For example, in our data set, the NK-enhanced SDIEALGA variant of RI10953 showed reduced NK activity when combined with a variant that has putatively relatively low FcyR3a engaging activity such as IgG1 wildtype, C1q-enhancing K326W or FcRn-enhanced SELF variants (Supplementary Figure 5). In addition, the clone, i.e., the Fab portion, itself may directly influence the binding affinities to different Fcγ receptors. Along these lines, polymorphic variants of human Fcγ receptors do have different binding affinities for different Fc variants, adding to the complexity of the overall functional activity observed^21^ (Supplementary Figure 6). Thus, the complex interplay of antibody clone (i.e., Fab portion), antigen (i.e., binding epitope), and host factors (i.e., Fc receptor polymorphism) mutually determines the overall Fc activity.

There are several limitations to this study. Functionalities of Fc-modified antibody variants were all tested and compared in *in vitro* assays and, while the strict standardization of these assays allowed comparability between Fc variants and the different clones, it might differ from what might be observed *in vivo* where, for example, different effector cell availabilities, Fc receptor polymorphism, or local inflammatory milieu are likely to affect the efficacy of certain Fc modifications. ADNP and ADNKA assays were performed with primary cells. While blood donors were randomly selected, we did not genotype for Fc receptor polymorphism and our analysis might be biased toward certain genotypes.^43^ NK cell activation was approximated, as previously reported by us and other groups,^44–46^ by the expression of the degranulation marker CD107a and cytokines IFNγ and MIP-1β while future analysis will need to confirm direct ADCC. Furthermore, for feasibility reasons we performed pairwise functional comparisons of the different Fc-modified variants at a single antibody concentration and more subtle differences due to antibody stoichiometry might have been missed. Finally, all antibodies were produced in the same HEK293 cell line and therefore equipped with a similar Fc-glycosylation signature. Future studies will need to explore how glycan changes coupled with Fc-mutational modifications will affect antibody-mediated functions.

In conclusion, these data provide initial evidence of augmented Fc-mediated activities in antibody combinations that have been equipped with complementary Fc modifications. We observed, however, that more than half of the tested Fc modifications induced differing magnitudes of Fc-induced functionalities between the two mAb specificities, clearly indicating an impact of epitope on function. Furthermore, certain Fc variants had detrimental effects when combined, suggesting that the functional outcome of combinations is not easily predicted. This might be explained by differences in Fc accessibility across different epitopes and allosteric competition in binding. Likewise, context-dependent and cell-dependent FcyR expression patterns on effector cells will impact their sensitivity to Fc-modified antibodies. Careful selection of combinatorial Fc modifications to fine tune their activity and tailor it to the therapeutic goals will be critical and future studies will need to determine ideal Fc modifications for optimal efficacy *in vivo*.

## Materials and Methods

### Antibody cloning and production

Variable heavy and light chain sequences of anti-HIV mAbs RI808 and RI10953 were described previously. Unique 5’ leader and 3’ tail sequences were added to both variable domain sequences and shuttled into pUC plasmids. By the Golden Gate cloning^47^ approach, the variable domain plasmids were combined with plasmids containing “Golden Gate cloning ready” P2A sequence, lambda light chain sequence and sequences of the different Fc variants, respectively, along with a type IIs restriction enzyme and T4 DNA ligase to generate mammalian expression vectors.^14^ Sequences of the production plasmid inserts were confirmed by Sanger sequencing and plasmids amplified in *E. coli* and purified by Mini-Prep. HEK293F suspension cells cultured in FreeStyle 293 Expression medium were transfected with the respective production plasmid using polyethyleneimine (PEI). Supernatants were harvested 7 days post transfection and antibodies purified by Protein G magnetic beads (New England Biolabs). Upon glycine elution (TRIS buffered) mAbs were immediately buffer exchanged to phosphate-buffered saline (PBS) and concentrated using Amicon centrifugation filters (50 kDa cutoff). Antibody concentration was then measured by bicinchoninic acid assay. The ability to bind to HIV gp120 antigen (Immune Technology Corp.: Cat. No. IT-001-0025p) and purity was confirmed by HIV gp120-specific enzyme-linked immunosorbent assay (ELISA) and SDS-PAGE, respectively. All produced variants were able to bind to gp120 in the ELISA and only showed antibody specific/expected protein bands in the SDS-PAGE analysis. Antibodies were stored at 4°C for up to four weeks. Before use, antibodies were spun down 10,000×g for 10 min at 4°C and supernatants collected to remove possible aggregates. Concentrations of supernatants were confirmed by A280 measurement on a Nanodrop instrument.

### Fc receptor binding

The ability of antibodies to interact with FcγR was evaluated using a Luminex-bead based assay (Luminex Corp, Austin, TX, USA), as previously described.^48^ Biotinylated antigen was coupled to Luminex beads. These beads were then incubated with antibodies to form immune-complexes. PE-streptavidin (Agilent Technologies, Santa Clara, CA, USA) coupled to recombinant human FcγR protein (containing a C-terminal biotinylated AVI-tag; produced at the Duke Human Vaccine Institute) was used as the secondary probe. After 1 hour of incubation, samples were washed, and relative Fc receptor binding levels quantified using an iQue analyzer (IntelliCyt, Albuquerque, NM, USA). All FcγR binding levels are reported as median fluorescence intensity (MFI).

### Antibody-dependent cellular phagocytosis

Biotinylated gp120 antigen was incubated with 1 µm yellow-green fluorescent neutravidin beads (Invitrogen) overnight. 300 ng mAbs were mixed with antigen-labeled beads and incubated for 2 hours. THP-1 cells (2.5 × 10^4^ cells per well; ATCC: Cat. No. TIB-202) were then added and incubated overnight under standard tissue culture conditions. After incubation, the co-cultures were fixed and analyzed on a IQue3 analyzer. For analysis, the samples were gated on cells using forward scatter and side scatter, and the proportion of THP-1 cells phagocytosing beads was determined. The data represents the results of two separate experiments for which a phagocytic score was calculated as follows: (% bead positive x MFI bead positive).^49^

### Antibody-dependent neutrophil phagocytosis

Biotinylated gp120 antigen was incubated with 1 µm yellow-green fluorescent neutravidin beads (Invitrogen) overnight. 1000 ng mAbs were mixed with antigen-labeled beads and incubated for 2 hours. Primary human neutrophils (5.0 × 10^4^ cells per well) isolated by ammonium – chloride – potassium lyses from EDTA blood were then added and incubated for 1 hour under standard tissue culture conditions. After incubation, cells were stained with anti-human CD66b (clone: G10F5, Biolegend: Cat. No. 305112), were fixed and analyzed on a IQue3 analyzer. For analysis, the samples were gated on CD66b+ cells, and the proportion of primary human neutrophils phagocytosing beads was determined. The data represents the results of two separate experiments for which a phagocytic score was calculated as follows: (% bead positive × MFI bead positive).^50^ Whole blood samples for neutrophil isolation were collected at the Ragon Institute of MGH, MIT and Harvard. All donors were 18 years or older and gave signed consent. Samples were deidentified before use, and the study was approved by the MGH Institutional Review Board.

### Antibody-dependent complement deposition

Biotinylated gp120 antigen was incubated with 1 µm yellow-green fluorescent neutravidin beads (Invitrogen) overnight. 1000 ng mAbs were mixed with antigen-labeled beads and incubated for 2 hours. Guinea pig complement factor (Cedarlane: Cat. No. CL4051) in GVB++ buffer was added to the immune complexes and incubated for 20 minutes at 37°C. The complement reaction was then stopped by two washes with 5 mM EDTA in PBS. Complement factor C3 deposition on beads was then determined by an anti-guinea pig C3 antibody (polyclonal, MP Biomedicals: Cat. No. 0855385). Stained beads were fixed and analyzed on a IQue3 analyzer.

### Antibody-dependent NK cell activation

ELISA plates (Thermo Fisher) were coated with nonbiotinylated gp120 antigen and then blocked. 500 ng mAbs were added to each well. NK cells were isolated from buffy coats from healthy donors using the RosetteSep NK cell enrichment kit (STEMCELL Technologies, MA, USA) and stimulated with human recombinant IL-15 (rhIL-15) (1 ng/ml; STEMCELL Technologies) at 37°C overnight. NK cells were added to the ELISA plate and incubated together with anti-CD107a (clone H4A3, BD: Cat. No. 555802), brefeldin A (Sigma-Aldrich, MO, USA), and monensin (BD) for 5 h at 37°C. Next, cells were surface stained for CD56 (clone B159, BD Cat. No. 557747), and CD3 (clone UCHT1, BD: Ca. no. 557943). After fixation and permeabilization with FIX & PERM cell permeabilization kit (Thermo Fisher), cells were stained for intracellular markers MIP-1β (clone D21–1351, BD: Cat. No. 550078) and IFN-γ (clone B27, BD: Cat. No. 554702). NK cells were defined as CD3-CD56+ and frequencies of degranulated (CD107a+), IFN-γ+, and MIP1β+ NK cells determined by flow cytometry on a IQue3 analyzer. Buffy coats for NK cell preparation from healthy volunteers were collected and processed by the Massachusetts General Hospital (MGH) Blood Bank. All donors were 18 years or older and gave signed consent. Samples were deidentified before use, and the study was approved by the MGH Institutional Review Board.

### Experimental design of the assays

To determine the optimal concentration for each functional assay, we first tested unmutated IgG1 of RI808. For each assay we then selected an assay-specific antibody concentration (the same for both Fab clones) at which we observed a detectable but low signal. Since we wanted to focus on augmentation of antibody functionality by Fc mutation by selecting a low antibody concentration, we increased the detection range to assess functional augmentation. ADCP and ADCD assays were performed with two technical replicates for each mAb or mAb combination. Biological replicates (as individual blood donors) were performed for ADNP (two donors) and ADNKA (at least three donors) assay (Supplementary Figure 2 and 3). Experiments of each Fab clone or combination experiments were performed on the same day with the same cell donors to account for biological variability. Average of replicates was used for the analyses. Negative controls (PBS and pooled normal human IgG [IVIG]) were included in each assay. Assays were only called valid if replicates were correlated (*r* ≥ 0.7; except ADNKA) and values for controls followed the expected patterns.

### Data analyses

Data analysis was performed on GraphPad Prism (v.9.3) and R (v.4.0.1) and RStudio (v.1.3). For the analysis of the different variants and combinations the data was Z-Scored. PCA and k-means clustering was performed and visualized in R using the factoextra package (v.1.0.7). Heatmaps were generated with the pheatmap package (v.1.0.12). Flower plots were visualized with the ggplot2 package (v.3.3) using Z-Scored data (Z-Score was calculated per clone and Fc function, respectively). An F-Score was calculated to assess the functional NK vs. phagocytic activity of the Fc variants. F-Score was defined as the difference of the sum of phagocytic functions (ADNP and ADCP) and the sum of NK features. To evaluate the performance of heterologous Fc variant combinations, we first derived an expected value for each Fc function based on the non-combined values. Thus, the expected value was the sum of the two non-combined clone variants at half concentration. This expected value was then subtracted from the observed value. Using the difference values, a Z-Score was calculated for each Fc effector function analyzed and the combinations were ranked by their Z-Score (negative Z-scores indicate a lower than expected observed value and positive Z-scores indicate a higher than expected value).

## Supplementary Material

Supplemental Material

supp figures.zip

## References

[1] Carter PJ, Rajpal A. Designing antibodies as therapeutics. Cell. 2022;185(15):2789–10. doi:10.1016/j.cell.2022.05.029. 35868279

[2] Ahmed AA, Keremane SR, Vielmetter J, Bjorkman PJ. Structural characterization of GASDALIE Fc bound to the activating Fc receptor FcγRIIIa. J Struct Biol. 2016;194(1):78–89. doi:10.1016/j.jsb.2016.02.001. 26850169 PMC4769027

[3] Shields RL, Namenuk AK, Hong K, Meng YG, Rae J, Briggs J, Xie D, Lai J, Stadlen A, Li B, et al. High resolution mapping of the binding site on human IgG1 for FcγRI, FcγRII, FcγRIII, and FcRn and Design of IgG1 Variants with Improved Binding to the FcγR. J Biol Chem. 2001;276(9):6591–6604. doi:10.1074/jbc.M009483200. 11096108

[4] Horton HM, Bernett MJ, Pong E, Peipp M, Karki S, Chu SY, Richards JO, Vostiar I, Joyce PF, Repp R, et al. Potent in vitro and in vivo activity of an Fc-engineered anti-CD19 monoclonal antibody against lymphoma and leukemia. Cancer Res. 2008;68(19):8049–8057. doi:10.1158/0008-5472.CAN-08-2268. 18829563

[5] Awan FT, Lapalombella R, Trotta R, Butchar JP, Yu B, Benson DM Jr., Roda JM, Cheney C, Mo X, Lehman A, et al. CD19 targeting of chronic lymphocytic leukemia with a novel Fc-domain–engineered monoclonal antibody. Blood. 2010;115(6):1204–1213. doi:10.1182/blood-2009-06-229039. 19965644 PMC2826232

[6] Bang YJ, Giaccone G, Im SA, Oh DY, Bauer TM, Nordstrom JL, Li H, Chichili GR, Moore PA, Hong S, et al. First-in-human phase 1 study of margetuximab (MGAH22), an Fc-modified chimeric monoclonal antibody, in patients with HER2-positive advanced solid tumors. Ann Oncol. 2017;28(4):855–861. doi:10.1093/annonc/mdx002. 28119295 PMC6246722

[7] Lu LL, Chung AW, Rosebrock TR, Ghebremichael M, Yu WH, Grace PS, Schoen MK, Tafesse F, Martin C, Leung V, et al. A functional role for antibodies in tuberculosis. Cell. 2016;167(2):433–43 e14. doi:10.1016/j.cell.2016.08.072. 27667685 PMC5526202

[8] Zhang A, Stacey HD, Mr D, Tugg Y, Marzok A, Miller MS. Beyond neutralization: Fc-dependent antibody effector functions in SARS-CoV-2 infection. Nat Rev Immunol. 2023;23(6):381–396. doi:10.1038/s41577-022-00813-1. 36536068 PMC9761659

[9] Rossignol E, Alter G, Julg B. Antibodies for human immunodeficiency virus-1 cure strategies. J Infect Dis. 2021;223(12 Suppl 2):22–31. doi:10.1093/infdis/jiaa165. 33586772 PMC7883024

[10] Bartsch YC, Cizmeci D, Kang J, Zohar T, Periasamy S, Mehta N, Tolboom J, Van der Fits L, Sadoff J, Comeaux C, et al. Antibody effector functions are associated with protection from respiratory syncytial virus. Cell. 2022;185(26):4873–86 e10. doi:10.1016/j.cell.2022.11.012. 36513064

[11] Atyeo C, Slein MD, Fischinger S, Burke J, Schafer A, Leist SR, Kuzmina NA, Mire C, Honko A, Johnson R, et al. Dissecting strategies to tune the therapeutic potential of SARS-CoV-2–specific monoclonal antibody CR3022. JCI Insight. 2021;6(1). doi:10.1172/jci.insight.143129. PMC7821590 33427208

[12] Wagh K, Seaman MS. Divide and conquer: broadly neutralizing antibody combinations for improved HIV-1 viral coverage. Curr Opin HIV AIDS. 2023;18(4):164–170. doi:10.1097/COH.0000000000000800. 37249911 PMC10256304

[13] Rossignol ED, Dugast AS, Compere H, Cottrell CA, Copps J, Lin S, Cizmeci D, Seaman MS, Ackerman ME, Ward AB, et al. Mining HIV controllers for broad and functional antibodies to recognize and eliminate hiv-infected cells. Cell Rep. 2021;35(8):109167. doi:10.1016/j.celrep.2021.109167. 34038720 PMC8196545

[14] Gunn BM, Lu R, Slein MD, Ilinykh PA, Huang K, Atyeo C, Schendel SL, Kim J, Cain C, Roy V, et al. A Fc engineering approach to define functional humoral correlates of immunity against ebola virus. Immunity. 2021;54(4):815–28 e5. doi:10.1016/j.immuni.2021.03.009. 33852832 PMC8111768

[15] Zalevsky J, Chamberlain AK, Horton HM, Karki S, Leung IW, Sproule TJ, Lazar GA, Roopenian DC, Desjarlais JR. Enhanced antibody half-life improves in vivo activity. Nat Biotechnol. 2010;28(2):157–159. doi:10.1038/nbt.1601. 20081867 PMC2855492

[16] Datta-Mannan A, Witcher DR, Tang Y, Watkins J, Jiang W, Wroblewski VJ. Humanized IgG1 variants with differential binding properties to the neonatal Fc receptor: relationship to pharmacokinetics in mice and primates. Drug Metab Dispos. 2007;35(1):86–94. doi:10.1124/dmd.106.011734. 17050651

[17] Idusogie EE, Presta LG, Gazzano-Santoro H, Totpal K, Wong PY, Ultsch M, Meng YG, Mulkerrin MG. Mapping of the C1q binding site on rituxan, a chimeric antibody with a human IgG1 Fc. J Immunol. 2000;164(8):4178–4184. doi:10.4049/jimmunol.164.8.4178. 10754313

[18] Idusogie EE, Wong PY, Presta LG, Gazzano-Santoro H, Totpal K, Ultsch M, Mulkerrin MG. Engineered antibodies with increased activity to recruit complement. J Immunol. 2001;166(4):2571–2575. doi:10.4049/jimmunol.166.4.2571. 11160318

[19] Diebolder CA, Beurskens FJ, de Jong RN, Koning RI, Strumane K, Lindorfer MA, Voorhorst M, Ugurlar D, Rosati S, Heck AJR, et al. Complement is activated by IgG hexamers assembled at the cell surface. Science. 2014;343(6176):1260–1263. doi:10.1126/science.1248943. 24626930 PMC4250092

[20] Moore GL, Chen H, Karki S, Lazar GA. Engineered Fc variant antibodies with enhanced ability to recruit complement and mediate effector functions. MAbs. 2010;2(2):181–189. doi:10.4161/mabs.2.2.11158. 20150767 PMC2840237

[21] Lazar GA, Dang W, Karki S, Vafa O, Peng JS, Hyun L, Chan C, Chung HS, Eivazi A, Yoder SC, et al. Engineered antibody Fc variants with enhanced effector function. Proc Natl Acad Sci USA. 2006;103(11):4005–4010. doi:10.1073/pnas.0508123103. 16537476 PMC1389705

[22] Richards JO, Karki S, Lazar GA, Chen H, Dang W, Desjarlais JR. Optimization of antibody binding to FcγRIIa enhances macrophage phagocytosis of tumor cells. Mol Cancer Ther. 2008;7(8):2517–2527. doi:10.1158/1535-7163.MCT-08-0201. 18723496

[23] Saito S, Namisaki H, Hiraishi K, Takahashi N, Iida S. A stable engineered human IgG3 antibody with decreased aggregation during antibody expression and low pH stress. Protein Sci. 2019;28(5):900–909. doi:10.1002/pro.3598. 30834577 PMC6459999

[24] Jendeberg L, Nilsson P, Larsson A, Denker P, Uhlen M, Nilsson B, Nygren P-Å. Engineering of Fc(1) and Fc(3) from human immunoglobulin G to analyse subclass specificity for staphylococcal protein a. J Immunol Methods. 1997;201(1):25–34. doi:10.1016/S0022-1759(96)00215-3. 9032407

[25] Steurer W, Nickerson PW, Steele AW, Steiger J, Zheng XX, Strom TB. Ex vivo coating of islet cell allografts with murine CTLA4/Fc promotes graft tolerance. J Immunol. 1995;155(3):1165–1174. doi:10.4049/jimmunol.155.3.1165. 7543517

[26] Lund J, Winter G, Jones PT, Pound JD, Tanaka T, Walker MR, Artymiuk PJ, Arata Y, Burton DR, Jefferis R, et al. Human Fc gamma RI and Fc gamma RII interact with distinct but overlapping sites on human IgG. J Immunol. 1991;147(8):2657–2662. doi:10.4049/jimmunol.147.8.2657. 1833457

[27] Stavenhagen JB, Gorlatov S, Tuaillon N, Rankin CT, Li H, Burke S, Huang L, Johnson S, Bonvini E, Koenig S. Fc optimization of therapeutic antibodies enhances their ability to kill tumor cells in vitro and controls tumor expansion in vivo via low-affinity activating Fcγ receptors. Cancer Res. 2007;67(18):8882–8890. doi:10.1158/0008-5472.CAN-07-0696. 17875730

[28] Walker MR, Lund J, Thompson KM, Jefferis R. Aglycosylation of human IgG1 and IgG3 monoclonal antibodies can eliminate recognition by human cells expressing FcγRI and/or FcγRII receptors. Biochem J. 1989;259(2):347–353. doi:10.1042/bj2590347. 2524188 PMC1138517

[29] Hinton PR, Johlfs MG, Xiong JM, Hanestad K, Ong KC, Bullock C, Keller S, Tang MT, Tso JY, Vásquez M, et al. Engineered human IgG antibodies with longer serum half-lives in primates. J Biol Chem. 2004;279(8):6213–6216. doi:10.1074/jbc.C300470200. 14699147

[30] Moldt B, Schultz N, Dunlop DC, Alpert MD, Harvey JD, Evans DT, Poignard P, Hessell AJ, Burton DR. A panel of IgG1 b12 variants with selectively diminished or enhanced affinity for Fcγ receptors to define the role of effector functions in protection against HIV. J Virol. 2011;85(20):10572–10581. doi:10.1128/JVI.05541-11. 21849450 PMC3187489

[31] Smith P, Dj D, Bournazos S, Li F, Ravetch JV. Mouse model recapitulating human Fcγ receptor structural and functional diversity. Proc Natl Acad Sci USA. 2012;109(16):6181–6186. doi:10.1073/pnas.1203954109. 22474370 PMC3341029

[32] Dall’acqua WF, Woods RM, Ward ES, Palaszynski SR, Patel NK, Brewah YA, Wu H, Kiener PA, Langermann S. Increasing the affinity of a human IgG1 for the neonatal Fc receptor: biological consequences. J Immunol. 2002;169(9):5171–5180. doi:10.4049/jimmunol.169.9.5171. 12391234

[33] Masuda K, Kubota T, Kaneko E, Iida S, Wakitani M, Kobayashi-Natsume Y, Kubota A, Shitara K, Nakamura K. Enhanced binding affinity for FcγRIIIa of fucose-negative antibody is sufficient to induce maximal antibody-dependent cellular cytotoxicity. Mol Immunol. 2007;44(12):3122–3131. doi:10.1016/j.molimm.2007.02.005. 17379311

[34] Chu SY, Vostiar I, Karki S, Moore GL, Lazar GA, Pong E, Joyce PF, Szymkowski DE, Desjarlais JR. Inhibition of B cell receptor-mediated activation of primary human B cells by coengagement of CD19 and FcγRIIb with Fc-engineered antibodies. Mol Immunol. 2008;45(15):3926–3933. doi:10.1016/j.molimm.2008.06.027. 18691763

[35] Lakerveld AJ, Gelderloos AT, Schepp RM, de Haan CAM, van Binnendijk RS, Rots NY, van Beek J, van Els CACM, van Kasteren PB. Difference in respiratory syncytial virus-specific Fc-mediated antibody effector functions between children and adults. Clin Exp Immunol. 2023;214(1):79–93. doi:10.1093/cei/uxad101. 37605554 PMC10711356

[36] Mackin SR, Desai P, Whitener BM, Karl CE, Liu M, Baric RS, Edwards DK, Chicz TM, McNamara RP, Alter G, et al. Fc-γR-dependent antibody effector functions are required for vaccine-mediated protection against antigen-shifted variants of SARS-CoV-2. Nat Microbiol. 2023;8(4):569–580. doi:10.1038/s41564-023-01359-1. 37012355 PMC10797606

[37] Gelderloos AT, Lakerveld AJ, Schepp RM, Nicolaie MA, van Beek J, Beckers L, van Binnendijk RS, Rots NY, van Kasteren PB. Primary SARS-CoV-2 infection in children and adults results in similar Fc-mediated antibody effector function patterns. Clin Transl Immunol. 2024;13(8):e1521. doi:10.1002/cti2.1521. PMC11273100 39071109

[38] Marchevsky NG, Li G, Aley P, Costa Clemens SA, Barrett JR, Belij-Rammerstorfer S, Bibi S, Clutterbuck E, Dold C, Felle S, et al. An exploratory analysis of the response to ChAdOx1 nCoV-19 (AZD1222) vaccine in males and females. EBioMedicine. 2022;81:104128. doi:10.1016/j.ebiom.2022.104128. 35779491 PMC9242842

[39] Brady T, Cayatte C, Roe TL, Speer SD, Ji H, Machiesky L, Zhang T, Wilkins D, Tuffy KM, Kelly EJ. Fc-mediated functions of nirsevimab complement direct respiratory syncytial virus neutralization but are not required for optimal prophylactic protection. Front Immunol. 2023;14:1283120. doi:10.3389/fimmu.2023.1283120. 37901217 PMC10600457

[40] Irvine EB, Nikolov A, Khan MZ, Peters JM, Lu R, Sixsmith J, Wallace A, van Woudenbergh E, Shin S, Karpinski W, et al. Fc-engineered antibodies promote neutrophil-dependent control of Mycobacterium tuberculosis. Nat Microbiol. 2024;9(9):2369–2382. doi:10.1038/s41564-024-01777-9. 39174703 PMC11371646

[41] Paciello I, Maccari G, Pantano E, Andreano E, Rappuoli R. High-resolution map of the Fc functions mediated by COVID-19-neutralizing antibodies. Proc Natl Acad Sci USA. 2024;121(3):e2314730121. doi:10.1073/pnas.2314730121. 38198525 PMC10801854

[42] Liu R, Oldham RJ, Teal E, Beers SA, Cragg MS. Fc-engineering for modulated effector functions—improving antibodies for cancer treatment. Antibodies (Basel). 2020;9(4):64. doi:10.3390/antib9040064. 33212886 PMC7709126

[43] Osborne JM, Chacko GW, Brandt JT, Anderson CL. Ethnic variation in frequency of an allelic polymorphism of human Fcγ RIIA determined with allele specific oligonucleotide probes. J Immunol Methods. 1994;173(2):207–217. doi:10.1016/0022-1759(94)90299-2. 8046255

[44] Bartsch YC, Loos C, Rossignol E, Fajnzylber JM, Yuan D, Avihingsanon A, Ubolyam S, Jupimai T, Hirschel B, Ananworanich J, et al. Viral rebound kinetics correlate with distinct HIV antibody features. mBio. 2021;12(2). doi:10.1128/mBio.00170-21. PMC8092214 33688003

[45] Ackerman ME, Mikhailova A, Brown EP, Dowell KG, Walker BD, Bailey-Kellogg C, Suscovich TJ, Alter G. Polyfunctional HIV-Specific antibody responses are associated with spontaneous HIV control. PloS Pathog. 2016;12(1):e1005315. doi:10.1371/journal.ppat.1005315. 26745376 PMC4706315

[46] Morrison BJ, Roman JA, Luke TC, Nagabhushana N, Raviprakash K, Williams M, Sun P. Antibody-dependent NK cell degranulation as a marker for assessing antibody-dependent cytotoxicity against pandemic 2009 influenza A(H1N1) infection in human plasma and influenza-vaccinated transchromosomic bovine intravenous immunoglobulin therapy. J Virol Methods. 2017;248:7–18. doi:10.1016/j.jviromet.2017.06.007. 28624584 PMC7113754

[47] Engler C, Kandzia R, Marillonnet S, El-Shemy HA. A one pot, one step, precision cloning method with high throughput capability. PLoS One. 2008;3(11):e3647. doi:10.1371/journal.pone.0003647. 18985154 PMC2574415

[48] Brown EP, Licht AF, Dugast AS, Choi I, Bailey-Kellogg C, Alter G, Ackerman ME. High-throughput, multiplexed IgG subclassing of antigen-specific antibodies from clinical samples. J Immunol Methods. 2012;386(1–2):117–123. doi:10.1016/j.jim.2012.09.007. 23023091 PMC3475184

[49] Ackerman ME, Moldt B, Wyatt RT, Dugast AS, McAndrew E, Tsoukas S, Jost S, Berger CT, Sciaranghella G, Liu Q, et al. A robust, high-throughput assay to determine the phagocytic activity of clinical antibody samples. J Immunol Methods. 2011;366(1–2):8–19. doi:10.1016/j.jim.2010.12.016. 21192942 PMC3050993

[50] Karsten CB, Mehta N, Shin SA, Diefenbach TJ, Slein MD, Karpinski W, Irvine EB, Broge T, Suscovich TJ, Alter G. A versatile high-throughput assay to characterize antibody-mediated neutrophil phagocytosis. J Immunol Methods. 2019;471:46–56. doi:10.1016/j.jim.2019.05.006. 31132351 PMC6620195

