# Supplementary material for: Combinatorial Fc modifications for complementary antibody functionality
Source: MAbs. 2025 Feb 14;17(1):2465391. doi: 10.1080/19420862.2025.2465391 (PMC11834420; doi:10.1080/19420862.2025.2465391)
Supplement: Supplemental Material [file KMAB_A_2465391_SM1294.docx]

**Supplementary Figures:**

**Supplemental Figure 1: Fc activity of RI808 and RI10953 on the unmodified (wt) human IgG1 backbone.** The two clones were evaluated for induction of A) ADCP, B) ADNP, C) ADCD, D) NK cell degranulation (%CD107a+ of NK cells), NK cell secretion of IFNγ (%IFNγ+ of NK cells), and NK secretion of MIP-1β (MIP-1β+ of NK cells) after immune complex formation with recombinant HIV Envelope glycoprotein. Bars represent the mean value and error bars the standard deviation of two independent replicates.

**Supplemental Figure 1 Alt-text:** Bar graphs plotting six different Fc effector functions for RI808 and RI10953. RI808 appears to have greater potential to mediate Fc effector functions.

**Supplemental Figure 2:** Fc effector functions for RI808 Fc variants (x-axis) were determined. ADCD and ADCP were performed using two technical replicates, ADNP was performed using two biological replicates (blood donors) and ADNKA was performed using five blood donors. Symbols indicate matching replicates or blood donors. Horizontal lines are the average. This figure is related to Figure 1.

**Supplemental Figure 2 Alt-text:** Six aligned scatter plots showing distribution of replicates across the different Fc variants. One plot corresponds to a specific assay.

**Supplemental Figure 3:** Fc effector functions for RI10953 Fc variants (x-axis) were determined. ADCD and ADCP were performed using two technical replicates, ADNP was performed using two biological replicates (blood donors) and ADNKA was performed using three blood donors. Symbols indicate matching replicates or blood donors. Horizontal lines are the average. This figure is related to Figure 1.

**Supplemental Figure 3 Alt-text:** Six aligned scatter plots showing distribution of replicates across the different Fc variants. One plot corresponds to a specific assay.

**Supplemental Figure 4: (**A-D) Fc receptor binding patterns Binding patterns of distinct RI808 Fc variants without (“naïve”, purple) or with (yellow) additional LS (MLNS) mutation. Arrows connecting same Fc variants.

**Supplemental Figure 4 Alt-text: (**A-D) Data points connected by arrows showing that same LS carrying variants have weaker interaction with Fc receptors.

**Supplemental Figure 5: Homologous combination of engineered mAbs can modulate Fc activity.** (A) The flower plots summarize the functions data of the respective combination (variants of RI808 in panel (A), and RI10953 in panel (B)). Each petal represents the average of the Z-scored value for the indicated feature. Assay-specific total antibody amounts for non-combined Fc variants (diagonal) were the same as the total antibody amounts used for the variant combinations.

**Supplemental Figure 5A Alt-text:** Flower charts of as circular plots plot each of the six measured Fc effector functions. For different Fc variant combinations of Fab clones RI808 an individual flower plot is depicted.

**Supplemental Figure 5B Alt-text:** Flower charts of as circular plots plot each of the six measured Fc effector functions. For different Fc variant combinations of Fab clones RI10953 an individual flower plot is depicted.

**Supplemental Figure 6:** The heatmap summarizes the (Z-Scored) Fc receptor binding of different RI808 Fc variants (rows). Binding was determined to FcγR2a variants R131 and H131, FcγR2b, FcγR3a variants V158 and F158, and FcγR3b (column). Binding interaction is indicated by the color key (red higher interaction/binding, blue weaker interaction/binding).

**Supplemental Figure 6 Alt-text:** Heatmap with blue and red tiles pointing to differential binding patterns of different Fc variants.
